# Role of VEGFR‐1 in melanoma acquired resistance to the BRAF inhibitor vemurafenib

**DOI:** 10.1111/jcmm.14755

**Published:** 2019-11-23

**Authors:** Maria Grazia Atzori, Claudia Ceci, Federica Ruffini, Mauro Trapani, Maria Luisa Barbaccia, Lucio Tentori, Stefania D'Atri, Pedro Miguel Lacal, Grazia Graziani

**Affiliations:** ^1^ Department of Systems Medicine University of Rome Tor Vergata Rome Italy; ^2^ Laboratory of Molecular Oncology IDI‐IRCCS Rome Italy

**Keywords:** angiogenesis, BRAF inhibitors, melanoma, VEGF‐A, VEGFR‐1, vemurafenib

## Abstract

The vascular endothelial growth factor receptor‐1 (VEGFR‐1) is a tyrosine kinase receptor frequently expressed in melanoma. Its activation by VEGF‐A or placental growth factor (PlGF) promotes tumour cell survival, migration and invasiveness. Moreover, VEGFR‐1 stimulation contributes to pathological angiogenesis and induces recruitment of tumour‐associated macrophages. Since melanoma acquired resistance to BRAF inhibitors (BRAFi) has been associated with activation of pro‐angiogenic pathways, we have investigated VEGFR‐1 involvement in vemurafenib resistance. Results indicate that human melanoma cells rendered resistant to vemurafenib secrete greater amounts of VEGF‐A and express higher VEGFR‐1 levels compared with their BRAFi‐sensitive counterparts. Transient VEGFR‐1 silencing in susceptible melanoma cells delays resistance development, whereas in resistant cells it increases sensitivity to the BRAFi. Consistently, enforced VEGFR‐1 expression, by stable gene transfection in receptor‐negative melanoma cells, markedly reduces sensitivity to vemurafenib. Moreover, melanoma cells expressing VEGFR‐1 are more invasive than VEGFR‐1 deficient cells and receptor blockade by a specific monoclonal antibody (D16F7 mAb) reduces extracellular matrix invasion triggered by VEGF‐A and PlGF. These data suggest that VEGFR‐1 up‐regulation might contribute to melanoma progression and spreading after acquisition of a drug‐resistant phenotype. Thus, VEGFR‐1 inhibition with D16F7 mAb might be a suitable adjunct therapy for VEGFR‐1 positive tumours with acquired resistance to vemurafenib.

## 1 INTRODUCTION

The vascular endothelial growth factor receptor‐1 (VEGFR‐1) is a membrane tyrosine kinase receptor for VEGF‐A, VEGF‐B and placental growth factor (PlGF) that are all members of the VEGF family of angiogenic factors.^1^ At variance with VEGF‐A that also binds to VEGFR‐2 even though with lower affinity, VEGF‐B and PlGF are exclusive ligands for VEGFR‐1.^2^ This receptor is expressed in endothelial, smooth muscle cells and monocytes/macrophages, promoting chemotaxis and survival.^3^ In particular, VEGFR‐1 stimulation is implicated in pathological angiogenesis and induces the recruitment of tumour‐associated macrophages that, in turn, favour cancer progression and dissemination.^4^, ^5^, ^6^ In addition to the membrane receptor, soluble VEGFR‐1 forms have been identified, which derive from alternative splicing of the pre‐mRNA.^7^ The soluble receptor is released in the extracellular matrix (ECM) and exerts anti‐angiogenic effects by sequestering VEGF‐A or PlGF, thus lowering their availability for membrane receptor activation, or by forming dominant negative complexes via heterodimerization with the membrane‐associated receptors.

The VEGFR‐1 expression has been frequently reported also in tumour cells of different tissue origin.^1^ In melanoma cells, VEGFR‐1 activation by autocrine or paracrine ligands promotes tumour cell survival, migration, invasiveness and chemoresistance.^8^, ^9^, ^10^, ^11^, ^12^, ^13^, ^14^ Moreover, VEGFR‐1 is involved in vasculogenic mimicry, which provides cancer cells with oxygen and nutrient supply and a route for metastatic spreading.^14^, ^15^, ^16^ Using a recently developed monoclonal antibody (mAb), we have demonstrated that blockage of VEGFR‐1 activation inhibits neovessel formation, myeloid progenitor mobilization, melanoma infiltration by monocytes/macrophages and vasculogenic mimicry.^1^, ^14^ The D16F7 mAb is characterized by an innovative mechanism of action, by which it down‐modulates membrane receptor signalling without hampering VEGF‐A or PlGF ligand binding.^1^, ^14^ Based on this property, the D16F7 mAb does not interfere with the decoy function of the soluble VEGFR‐1 maintaining its anti‐angiogenic effects.

Malignant melanoma is a highly aggressive malignancy and is regarded as the most lethal form of cutaneous cancer because of its ability to metastasize to distant organs. The approval of immune checkpoints inhibitors (ie the anti‐PD‐1 and anti‐CTLA‐4 mAbs) and, for BRAF mutated melanoma (approximately 50% of all cases), of BRAF/MEK kinase inhibitors has dramatically improved the outcome of patients with metastases.^17^, ^18^ Unfortunately, in the case of immune checkpoint inhibitors a significant proportion of patients derive no benefit from these therapies (primary resistance), whereas in the case of BRAF/MEK inhibitors most patients respond to treatment but responses are short‐lived because of the development of drug resistance and tumour recurrence. Besides genetic alterations that result in reactivation of the MAPK and, less frequently, activation of the PI3K‐Akt pathways, other mechanisms are involved in acquired resistance to BRAF inhibitors (BRAFi) including activation of pro‐angiogenic pathways.^19^, ^20^ In this regard, the onset of treatment resistance to the BRAFi dabrafenib is associated with restored VEGF‐A production by melanoma cells.^21^, ^22^, ^23^ Moreover, by paradoxically activating the MAPK pathway in BRAF wild‐type macrophages, BRAFi may induce the production of VEGF‐A, which directly stimulates macrophage survival, tumour immune evasion and ultimately melanoma growth.^20^, ^24^, ^25^ Conversely, treatment of susceptible BRAF mutated melanoma with BRAFi results in reduced tumour vascularity and increased T cell infiltration in melanoma that was attributed to loss or reduced VEGF‐A expression and secretion.^26^, ^27^, ^28^

In the present study, we have investigated whether VEGFR‐1 might contribute to the acquisition of a BRAFi‐resistant phenotype by melanoma and whether blockade of this receptor might reduce ECM invasion by resistant tumour cells in response to angiogenic factors. The results indicate that human melanoma cells rendered resistant to the BRAFi vemurafenib express higher levels of VEGFR‐1 compared to their BRAFi‐sensitive counterparts and that inhibition of VEGFR‐1 with D16F7 mAb might be a suitable adjunct therapy for VEGFR‐1 positive tumours with acquired resistance to vemurafenib.

## 2 MATERIALS AND METHODS

### 2.1 Cell lines and culture conditions

The human melanoma A375 cell line was obtained from American Type Culture Collection (ATCC). The human melanoma M14C2 clone (hereafter referred to as M14) was obtained by limiting dilution from the corresponding bulk cell population, as previously described.^12^ The human melanoma GR‐Mel cell line was established in the Laboratory of Molecular Oncology, IDI‐IRCCS (Rome, Italy). A375 and M14 melanoma cell lines with acquired resistance to vemurafenib, hereafter referred to as A375‐VR and M14‐VR, respectively, were generated in our laboratories by exposing the parental cell line to increasing concentrations of vemurafenib (up to 5 µmol/L) for 3 months.

Cells were maintained in RPMI (Sigma‐Aldrich, St. Louis, MO, USA) supplemented with 10% foetal bovine serum (FBS, Sigma‐Aldrich), 2 mmol/L L‐glutamine, 100 units/mL penicillin and 100 μg/mL streptomycin sulphate, at 37°C in a 5% CO_2_ humidified atmosphere. Vemurafenib‐resistant cells were maintained in the presence of 2.5 µmol/L vemurafenib.

### 2.2 VEGFR‐1 stable transfection in melanoma cells

Generation of stable M14 subclones overexpressing VEGFR‐1 were obtained by cell transfection with the pBLAS49.2/VEGFR‐1 plasmid; control cells were transfected with the empty vector. The pBLAS49.2/VEGFR‐1 construct was obtained by cloning of VEGFR‐1 cDNA from the pcDNA3/VEGFR‐1 plasmid (a generous gift of Dr K. Ballmer‐Hofer, PSI, Zurich) into the pBLAS49.2 vector (InvivoGen). Transfection was performed using Lipofectamine 2000 (Invitrogen; ThermoFisher Scientific), as described by the manufacturer, and transfected cells were selected in blasticidine (Invitrogen) containing culture medium. Antibiotic resistant clones were isolated by ring cloning, and transfected clones maintained in the presence of 2.5 μg/mL blasticidine. VEGFR‐1 expressing subclones were identified by Western blotting.

### 2.3 ECM cell invasion assay

In vitro invasion assays were performed using Boyden chambers equipped with 8‐µm pore diameter polycarbonate filters (Nuclepore; Whatman Incorporated, Clifton, NJ), coated with 20 µg of matrigel, as described.^29^ Briefly, melanoma cells were suspended in invasion medium (1 µg/mL heparin/0.1% BSA in RPMI 1640) and loaded (2 × 10^5^ cells) into the upper compartment of the Boyden chambers. Invasion medium with or without VEGF‐A or PlGF (50 ng/mL) was added to the lower compartment of the chambers. Where indicated, invasion assays were performed in the presence of the anti‐VEGFR‐1 D16F7 mAb (5 µg/mL), after cell pre‐incubation with the mAb for 30 minutes at room temperature in a rotating wheel. For each experimental condition, three Boyden chambers were set up. After incubation at 37°C in a CO_2_ incubator for 4 hours, filters were removed and cells fixed in ethanol for 5 minutes and stained in 0.5% crystal violet for 15 minutes. Non‐invading cells were removed from the upper surface of the filter by wiping with a cotton swab and migrated cells, attached to the lower surface of the filters, were counted under the microscope. Twelve high‐magnification microscopic fields (200× magnification), randomly selected on triplicate filters, were scored for each experimental condition.

### 2.4 Cell proliferation assay

Cell proliferation was evaluated in 96‐well plates using the tetrazolium compound MTS [3‐(4,5‐dimethylthiazol‐2‐yl)‐5‐(3‐carboxymethoxyphenyl) 2‐(4‐sulphophenyl)‐2H–tetrazolium, inner salt] from Promega (Madison, WI, USA). Briefly, melanoma cells (800‐1000/well) were dispensed into flat‐bottom 96‐well plates and grown at 37°C in a 5% CO_2_ humidified atmosphere. For chemosensitivity assay, cells were exposed to graded concentrations of vemurafenib (PLX4032; Hoffmann‐La Roche Ltd, Basel, Switzerland). Vemurafenib was dissolved in DMSO and, just before use, diluted to the appropriate concentrations in complete medium with final DMSO concentration never exceeding 0.05% (v/v). Six replica wells were used for each condition in a total volume of 100 μL. After 5 days, 20 μL of MTS solution was added to each well and cells were incubated at 37°C for 1‐3 hours. Absorbance was read at 490 nm (reference wavelength 655 nm) using a 3550‐UV Microplate reader (Bio‐Rad, Hercules, CA, USA). Chemosensitivity was measured in terms of IC_50_, that is the concentration of the drug capable of inhibiting cell growth by 50%.

To evaluate cell doubling time, MTS was added to cells at different time‐points (0, 24, 48 and 72 h) after seeding (800‐1000 cells/well).

### 2.5 Analysis of VEGFRs transcripts

Quantification of membrane VEGFR‐1 and VEGFR‐2 transcripts was performed by quantitative real‐time reverse transcriptase‐polymerase chain reaction (qRT‐PCR) according to the dual‐labelled fluorigenic probe method and using an ABI Prism 7000 sequence detector (PerkinElmer, Groningen, the Netherlands) and using SYBR green master mix reagent, as previously described.^30^ Expression levels were calculated by the relative standard curve method. Primers, validated to specifically amplify human VEGFR‐1,^12^ were as follows: VEGFR‐1, forward 5′‐ACCGAATGCCACCTCCATG‐3′ and reverse 5′‐AGGCCTTGGGTTTGCTGTC‐3′; VEGFR‐2, forward 5′‐GTCTATGCCATTCCTCCCCC‐3′ and reverse 5′‐GAGACAGCTTGGCTGGGCT‐3′. For each sample, the level of VEGFR‐1 or VEGFR‐2 transcripts was normalized to that of 18S RNA (TaqMan^®^ Gene Expression Assay; Applied Biosystems) and referred to the values of the VEGFR‐1 and VEGFR‐2 negative M14 bulk cell line, to which the arbitrary value of 1 was assigned. A melting curve (62‐95°C) was generated at the end of each run to verify specificity of the reactions. The 2‐ΔΔCq relative quantification method was used to calculate mRNA expression.

### 2.6 ELISA quantification of VEGF‐A and PlGF levels

Conditioned media from melanoma cell lines were obtained by semi‐confluent cell cultures after incubation for 24 hours in 0.1% BSA/RPMI‐1640 medium without FBS. These conditions did not significantly affect cell viability. Culture supernatants were collected and concentrated at least 10‐fold in Centriplus concentrators (Amicon). Cells were detached from the flasks with a PBS/EDTA solution and the total cell number/culture was recorded to normalize cytokine secretion. The amounts of VEGF‐A and PlGF present in the culture medium were determined using commercial ELISA kits (R&D Systems), following the manufacturer's instructions. Optical density at 405 nm was measured in a 3550‐UV Microplate reader (Bio‐Rad).

### 2.7 Transient siRNA transfection

Melanoma cells were plated in complete medium, the day after transfected with 10 nmol/L siRNA directed against VEGFR‐1 (ID s535274; ThermoFisher Scientific) or AllStars Negative Control siRNA (siCTR; ID 1027281, Qiagen, Hilden, Germany) by using Lipofectamine RNAiMAX reagent (Invitrogen) and, after additional three days, analysed for VEGFR‐1 expression. For chemosensitivity assays, 24 hours after transfection, M14 and M14‐VR cells were exposed to DMSO alone or to graded concentrations of vemurafenib. Plates were incubated at 37°C for 5 days, and cell growth was evaluated by the MTS assay. Three replica wells were used for each group.

To analyse the influence of VEGFR‐1 on the development of resistance to vemurafenib, A375 cells (350 cells/well) were plated in triplicate into three BD Falcon^™^ 96‐well plates, allowed to adhere at 37°C for 18 hours, and then transfected with 10 nmol/L siRNA directed against VEGFR‐1 or siCTR, as described above. After 24 hours of culture, 200 nmol/L vemurafenib or DMSO was added to the wells. On day 7, the cells of one plate were fixed with ethanol and stained with 0.5% crystal violet. For quantitative analysis of proliferation, plates were photographed, images analysed by ImageJ software and results expressed as integrated density values. In the remaining plate, culture medium was changed and the cells were subjected to a new cycle of transfection and drug treatment as described above. After additional seven days (ie day 14), plates were fixed with ethanol, stained with 0.5% crystal violet and photographed to evaluate cell growth.

### 2.8 Immunoblot analysis

Proteins were run in 10% SDS‐polyacrylamide gels and transferred to supported nitrocellulose membranes by standard techniques. Membranes were incubated with the mouse monoclonal anti‐VEGFR‐1 (clone D2, 1:500; Santa Cruz Biotechnology), rabbit polyclonal anti‐Erk1&2 (1:1000; Genetex), rabbit polyclonal anti‐phospho‐Erk1&2 (Thr/Tyr185/187, 1:1000; Invitrogen) or rabbit polyclonal anti‐β‐tubulin (1:10 000; Santa Cruz Biotechnology) as primary antibodies. Immunodetection was performed using antimouse or anti‐rabbit Ig/Horseradish peroxidase secondary antibodies and ECL Western blotting detection reagents from GE Healthcare (Milan, Italy).

### 2.9 Statistical analysis

Statistical analysis of the differences between pairs of groups was performed by two‐sided Student's *t* test. For multiple comparisons, the non‐parametric Kruskal‐Wallis followed by Dunn's post hoc test was used. P values below 0.05 were considered statistically significant.

## 3 RESULTS

### 3.1 Generation and characterization of A375 and M14 sublines with acquired resistance to vemurafenib

**Figure 1:**
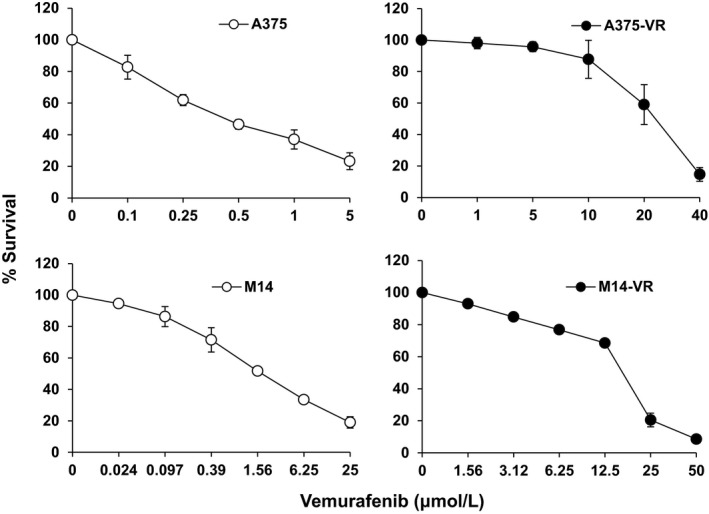
Antiproliferative effects of vemurafenib in A375 and M14 cells susceptible or rendered resistant to the BRAFi. Melanoma cells were incubated with increasing concentrations of vemurafenib or with the drug diluent (DMSO) alone for five days, and then, proliferation was assessed by the MTS assay. Data are expressed in terms of percentage of live cells relative to DMSO treated cells and are the arithmetic mean ± SD of three independent determinations

The vemurafenib‐resistant A375‐VR and M14‐VR melanoma cell lines were generated by chronic exposure of A375 and M14 cells, which harbour the BRAF V600E mutation and are susceptible to BRAFi,^31^ to increasing concentrations of vemurafenib. The doubling times, evaluated by MTS assay, for A375 and A375‐VR cells were 22.3 ± 3.6 h and 24.6 ± 5.9 h (*P* = .43), and for M14 and M14‐VR cells were 28.9 ± 4.2 h and 35.7 ± 4.5 h (*P* = .01), respectively. Analysis of chemosensitivity by MTS assay after 5 days of treatment indicated that the vemurafenib IC_50_ of A375‐VR cell line was ~31‐times higher than that of A375 parental cells (0.64 ± 0.1 µmol/L *vs* 20.2 ± 3.9 µmol/L, *P* < .0001), whereas in the case of M14‐VR it was ~11‐times higher than that of M14 parental cells (1.5 ± 0.4 µmol/L *vs* 16 ± 0.9 µmol/L, *P* < .0001; Figure 1).

### 3.2 Increased VEGFR‐1 expression and VEGF‐A secretion in melanoma cells resistant to vemurafenib

**Figure 2:**
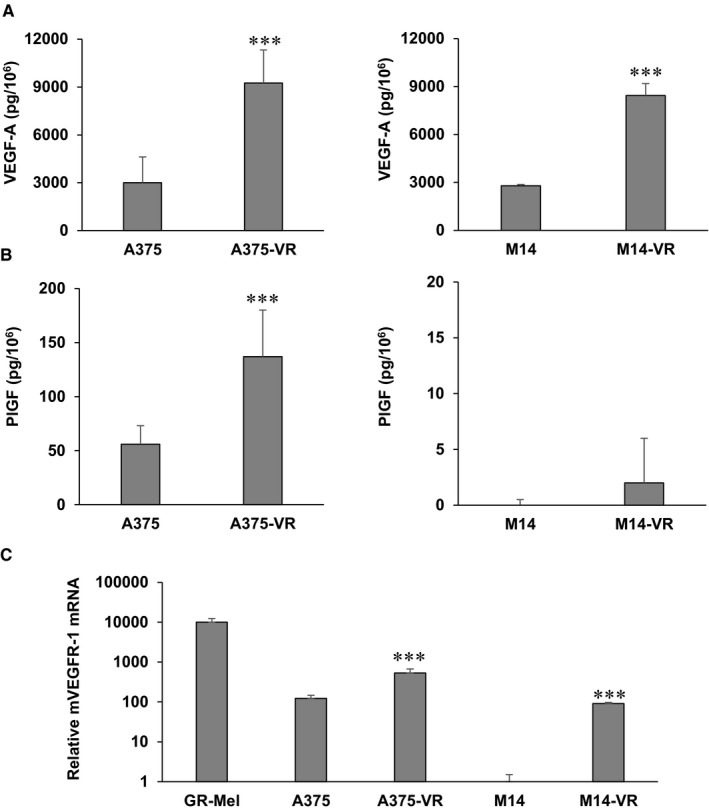
Characterization of human melanoma cell lines sensitive or resistant to vemurafenib for the production of VEGF‐A and PlGF and expression of VEGFR‐1. VEGF‐A (A) and PlGF (B) secretion was quantified by ELISA (mean ± SD; n = 3). Each value represents the arithmetic mean of three independent experiments performed with triplicate samples. C, The expression of VEGFR‐1 transcript was assessed by qRT‐PCR analysis utilizing the human melanoma GR‐Mel cell line as positive control. The results are expressed as relative mRNA and are the mean ± SD of three (A375 lines) or two (M14 lines) independent determinations with duplicate samples. Data were referred to the VEGFR‐1 negative M14 bulk cell line, to which the arbitrary value of 1 was assigned. Statistical analysis by two‐tailed Student's *t* test: resistant *vs* sensitive cells: ***P < .001

Vemurafenib susceptible and resistant melanoma cells were analysed for the production of VEGF‐A and PlGF in culture supernatants and for the expression of VEGFR‐1 and VEGFR‐2 by ELISA and qRT‐PCR, respectively. Both vemurafenib‐resistant melanoma A375‐VR and M14‐VR cells secreted higher VEGF‐A levels compared with control cells (Figure 2A). PlGF production was instead up‐modulated in A375‐VR compared with A375 cells, but it did not significantly change in M14‐VR compared with control M14 cells (Figure 2B). Concerning receptor transcript analysis in both A375‐VR and M14‐VR the VEGFR‐1 expression was significantly higher compared with their BRAFi susceptible counterparts (Figure 2C). Noteworthy, while A375 cells expressed basal VEGFR‐1 levels, M14 cells were negative for the expression of this receptor (Figure 2C). No detectable VEGFR‐2 transcript levels were observed in any of the vemurafenib‐sensitive and vemurafenib‐resistant cell lines (data not shown).

### 3.3 VEGFR‐1 silencing counteracts the emergence of resistance in sensitive cells and increases sensitivity to vemurafenib in resistant melanoma cells

**Figure 3:**
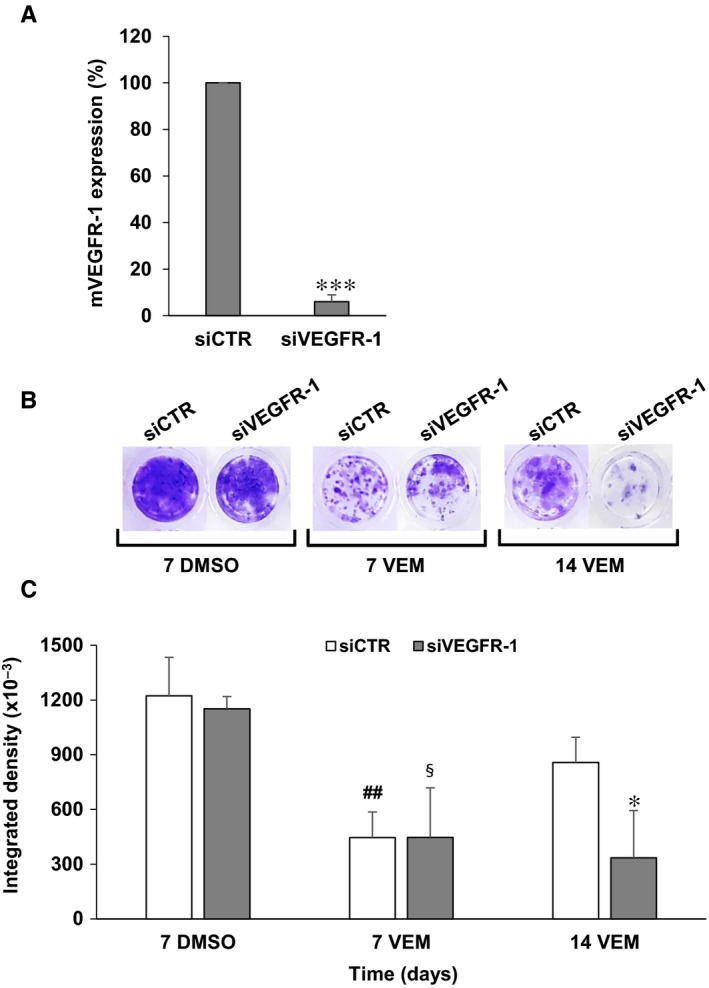
Influence of VEGFR‐1 silencing on the acquisition of resistance to vemurafenib by A375 cells. A, A375 cells were transfected with 10 nmol/L siVEGFR‐1 or siCTR and after three days total RNA was extracted and membrane VEGFR‐1 (mVEGFR‐1) transcript levels were assessed by qRT‐PCR analysis. Data are the mean of three independent determinations. Statistical analysis by two‐tailed Student's *t* test: ***P < .001. B, A375 cells (350/well) were seeded into 96‐well plates in triplicate, transfected with 10 nmol/L siVEGFR‐1 or siCTR and treated with 200 nmol/L vemurafenib (VEM) or the corresponding dilution of DMSO. Transfection was repeated after seven days. Cell cultures were stained in 0.5% crystal violet, photographed and processed for quantitative analysis of proliferation on day 7 and 14 after the first transfection. Images from a representative experiment are shown. C, Images were analysed by ImageJ software, and results were expressed as integrated density values. Each value represents the arithmetic mean of triplicate cultures. Statistical analysis by two‐tailed Student's *t* test: ^##^ *P* < .01, siCTR day 7 VEM *vs* siVEGFR‐1 day 7 DMSO; ^§^ *P* < .05, siVEGFR‐1 d 7 VEM *vs* siCTR day 7 DMSO; *P < .05, siVEGFR‐1 d 14 VEM *vs* siVEGFR‐1 day 14 VEM

To investigate the role of VEGFR‐1 in the acquisition of a vemurafenib‐resistant phenotype, A375 cells, which express basal levels of VEGFR‐1, were transiently silenced for the receptor and exposed to vemurafenib. Transfection of A375 cells with 10 nmol/L of a specific VEGFR‐1 siRNA (siVEGFR‐1) induced a marked reduction of the receptor transcript compared to transfection with a control siRNA (siCTR) (Figure 3A). A375 cells were then seeded into 96‐well plates and every seven days transfected with 10 nmol/L siVEGFR‐1 or siCTR and treated with 200 nmol/L vemurafenib or the equivalent volume of drug diluent (DMSO). A significant inhibition of cell proliferation was observed on day 7 after transfection in both control and VEGFR‐1 silenced cells exposed to vemurafenib (Figure 3B,C). However, while proliferation of drug‐treated siCTR/A375 cells resumed from day 7 to day 14, proliferation of drug‐treated siVEGFR‐1/A375 cells remained markedly inhibited, indicating a delay in the development of secondary resistance (Figure 3B,C).

**Figure 4:**
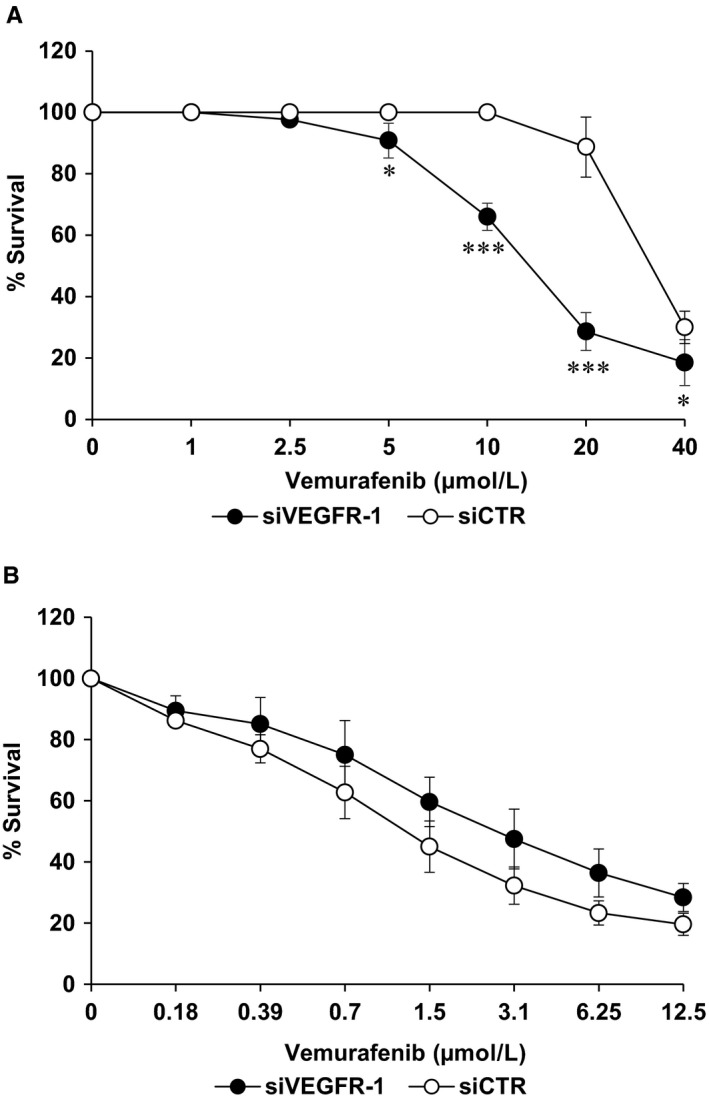
Influence of VEGFR‐1 silencing on the susceptibility to vemurafenib of M14‐VR and M14 cells. A and B, M14‐VR (A) or M14 (B) cells (1000/well) were seeded into 96‐well plates and the day after transfected with 10 nmol/L siVEGFR‐1 or siCTR and treated with graded concentrations of vemurafenib. After 5 d of culture, cell growth was analysed by MTS assay. Data are the mean of three independent experiments. Statistical analysis by two‐tailed Student's *t* test: *P < .05; ***P <0.001

Moreover, we have investigated the influence of VEGFR‐1 silencing on chemosensitivity to vemurafenib in M14‐VR melanoma cells, where acquisition of resistance to the BRAFi resulted in induction of the receptor that was instead absent in the parental cells. M14‐VR cells were seeded into 96‐well plates and transfected with 10 nmol/L siVEGFR‐1 or siCTR, treated with graded concentrations of vemurafenib and analysed by MTS assay after 7 days of culture. M14‐VR cells silenced for VEGFR‐1 showed a significant increase of susceptibility to vemurafenib compared with siCTR transfected cells (Figure 4A). In these experimental conditions, the IC_50_ value of M14‐VR cells transfected with siCTR resulted 31 ± 3.5 µmol/L, while that of M14‐VR cells silenced for VEGFR‐1 was 15.7 ± 1.0 µmol/L. Conversely, VEGFR‐1 silencing did not significantly affect the M14 cell susceptibility to the BRAFi (vemurafenib IC_50_ 1.6 ± 0.4 and 2.9 ± 0.86 in M14 cells transfected with siCTR or siVEGFR‐1, respectively; *P* = .11; Figure 4B).

### 3.4 Blockade of VEGFR‐1 inhibits ECM invasion by vemurafenib‐resistant melanoma cells

**Figure 5:**
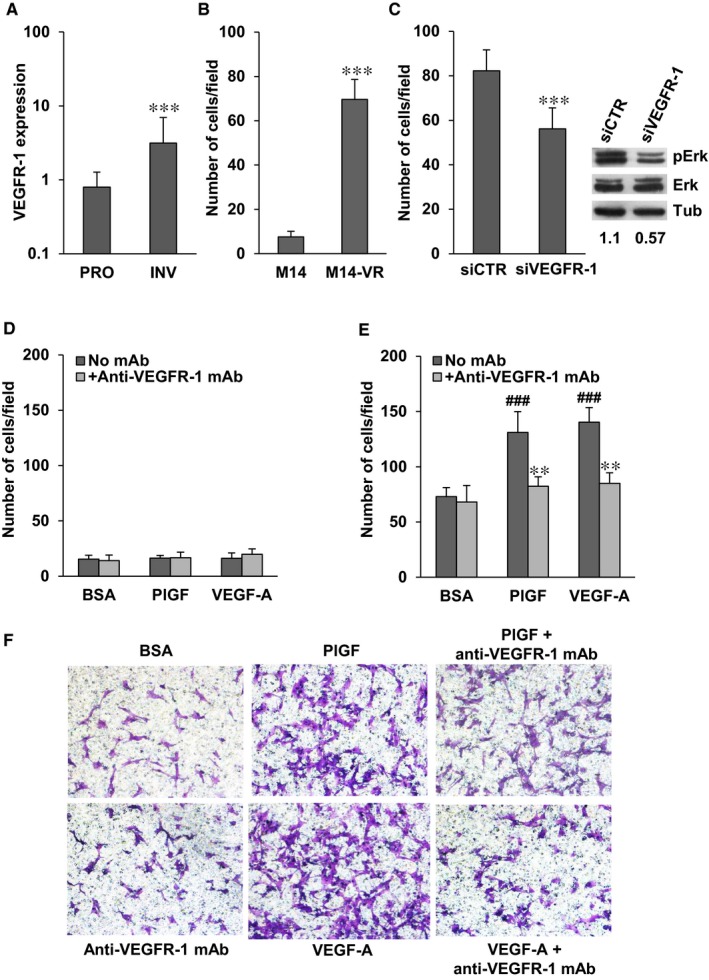
Expression of VEGFR‐1 in melanoma cells with proliferative or invasive phenotypes and inhibitory effect of the anti‐VEGFR‐1 mAb D16F7 on ECM invasion by M14‐VR melanoma cells in response to PlGF or VEGF‐A. A, HOPP analysis based on VEGFR‐1 expression levels was carried out using gene expression data sets including 189 melanoma cell lines and short‐term cultures, of which 100 are characterized by a proliferative phenotype and 89 by an invasive phenotype.^33^ Mean VEGFR‐1 transcript levels for proliferative (PRO) melanomas were compared with those of invasive melanomas (INV) and expressed as normalized signal intensity. Analysis of the 222033_s_at probeset for VEGFR‐1:3.9‐fold significant difference; statistical analysis by two‐tailed Student's *t* test: ***P < 1 × 10^−5^. B, Basal ECM invasion by M14 and M14‐VR cells (2 × 10^5^ cells/chambers, 4 h incubation) was evaluated in Boyden chambers equipped with matrigel‐coated filters. Statistical analysis by two‐tailed Student's *t* test: ***P < .001. C, ECM invasion by M14‐VR cells (2 × 10^5^ cells/chambers, 4 h incubation) transfected with siCTR or siVEGFR‐1. Statistical analysis by two‐tailed Student's *t* test: ***P < .001. Western blot (right panel) of phosphorylated Erk (pErk) and total Erk in M14‐VR cells transfected with siCTR or siVEGFR‐1; β‐tubulin was used as loading control. Numbers below blot lanes refer to densitometry measurements and indicate the ratios between optical densities of pErk and total Erk, after normalization for β‐tubulin expression. D and E, ECM invasion induced by PlGF (50 ng/mL) or VEGF‐A (50 ng/mL), in the absence or presence of 5 µg/mL anti‐VEGFR‐1 D16F7 mAb by M14 (D) or M14‐VR (E) cells. BSA, non‐stimulated cells. Histograms represent the arithmetic mean ± SD of invading cells/microscopic field from three independent experiments. Statistical analysis was performed by Kruskal‐Wallis followed by Dunn's test for multiple comparison: ^###^ *P* < .001, M14‐VR PlGF *vs* M14‐VR BSA and M14‐VR VEGF‐A *vs* M14‐VR BSA; **P < .05, M14‐VR PlGF *vs* M14‐VR PlGF + anti‐VEGFR‐1 mAb and M14‐VR VEGF‐A *vs* M14‐VR VEGF‐A + anti‐VEGFR‐1 mAb. F, Photographs from a representative experiment out of three are shown (×100 magnification)

According to the phenotype switching model, metastasis formation is the result of tumour transition from a proliferative to an invasive phenotype.^32^ An online gene expression‐based tool developed for predicting melanoma cell phenotype (ie Heuristic Online Phenotype Prediction, HOPP) is available and has identified a set of genes that characterizes these two different melanoma phenotypes.^33^ By using the HOPP algorithm, we have evaluated VEGFR‐1 expression in 220 melanoma cell lines and short‐term cultures grouped on the basis of their proliferative or invasive behaviour. Thirty‐one cell lines/cultures with both characteristics were excluded from the analysis. Taking into account a probe specific for the membrane VEGFR‐1, the expression of the receptor was significantly up‐modulated in the invasive melanoma group as compared to the highly proliferating group (Figure 5A). Consistently, induction of VEGFR‐1 expression in M14‐VR cells was associated with acquisition of an invasive phenotype as compared to the VEGFR‐1 negative M14 cells (Figure 5B). Moreover, A375 cells that expressed basal VEGFR‐1 levels showed ECM invasion also in the absence of specific receptor stimuli (data not shown). Transient silencing of VEGFR‐1 in M14‐VR cells caused a significant reduction of melanoma cell invasive ability that was accompanied by a decrease of Erk phosphorylation (Figure 5C).

**Figure 6:**
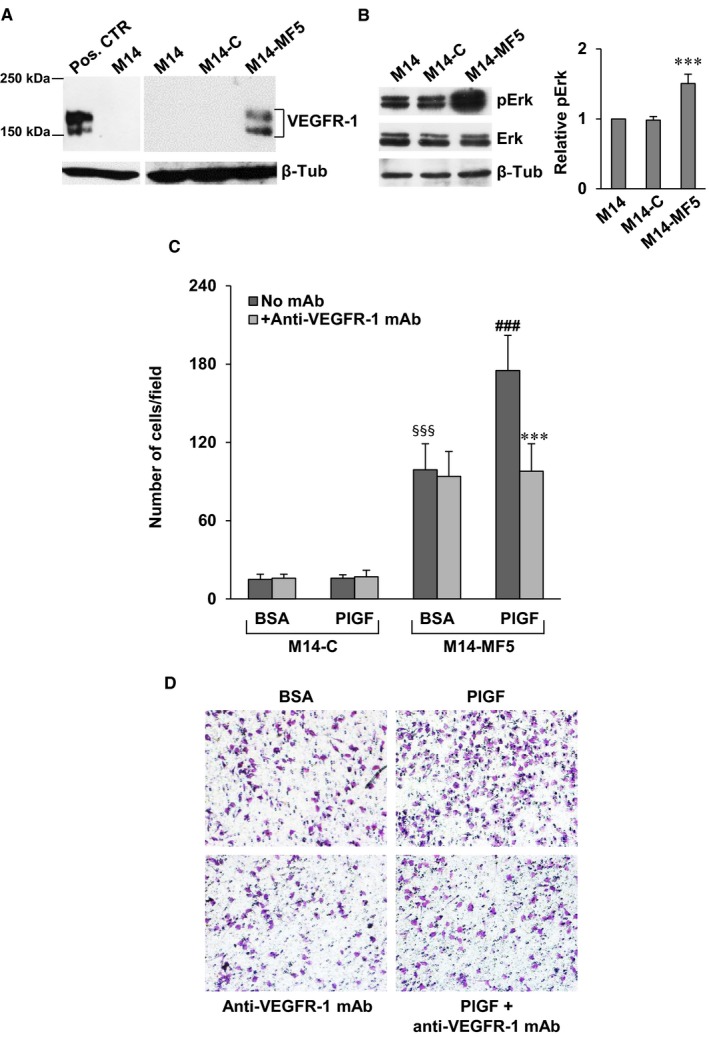
Enforced VEGFR‐1 expression in M14 melanoma cells increases invasiveness and reduces sensitivity to vemurafenib. A, VEGFR‐1 protein levels in M14 cells transfected with control (M14‐C) or VEGFR‐1 expressing (M14‐MF5) vectors were analysed by immunoblotting using antibodies against human VEGFR‐1 or β‐tubulin as loading control. The VEGFR‐1 protein has an expected molecular weight of 150 kD for the unmodified polypeptide and of 180‐185 kD for the glycosylated mature form. Positive control (Pos. CTR): glioblastoma cells transfected with the pBLAS49.2/VEGFR‐1 plasmid overexpressing the receptor.^42^ B, Western blot of phosphorylated Erk (pErk) and total Erk in M14, M14‐C and M14‐MF5 cells; β‐tubulin (β‐Tub) was used as loading control. Histogram represents the densitometric quantification of band intensities, expressed as pErk/Erk ratio relative to M14 non transfected cells, after normalization for β‐tubulin expression. Normalized pErk1/Erk protein ratio in M14 cells was considered equal to 1.Data are the mean ± SD of three independent experiments. Statistical analysis by two‐tailed Student's *t* test: ***P < .001. C, ECM invasion of M14‐C or M14‐MF5 cells (2 × 10^5^ cells/chamber, 2 h incubation) induced by PlGF (50 ng/mL), in the absence or presence of 5 µg/mL D16F7 mAb. BSA, non‐stimulated cells. Histograms represent the arithmetic mean ± SD of invading cells/microscopic field from three independent experiments. Statistical analysis was performed by Kruskal‐Wallis followed by Dunn's test for multiple comparison: *P* < .001, §§§, M14‐MF5 BSA *vs* M14‐C BSA; ###, M14‐MF5 PlGF *vs* M14‐MF5 BSA; ***, M14‐MF5 PlGF + anti‐VEGFR‐1 mAb *vs* M14‐MF5 PlGF. D, Photographs from a representative experiment with M14‐MF5 cells out of three are shown (×100 magnification)

On this basis, we have investigated whether pharmacological blockade of VEGFR‐1 by our recently developed D16F7 mAb might represent a suitable strategy to counteract invasiveness of receptor positive melanoma cells. Exposure of M14 cells, which lack VEGFR‐1 expression, to PlGF or VEGF‐A failed to induce matrigel invasion and treatment with D16F7 had no effect (Figure 5D). Conversely, PlGF and VEGF‐A stimulated ECM invasion by M14‐VR cells and D16F7 mAb efficiently counteracted (~75% inhibition) invasion triggered by the VEGFR‐1 ligands (Figure 5E,F). Moreover, enforced VEGFR‐1 overexpression in M14 cells by stable transfection of pBLAS49.2/VEGFR‐1 plasmid (Figure 6A) resulted in reduced sensitivity to vemurafenib. In fact, the BRAFi IC_50_ in the VEGFR‐1 overexpressing M14‐MF5 subclone was 11.6 ± 1.8 µmol/L, whereas in the subclone transfected with the control plasmid (M14‐C) the IC_50_ was 1.25 ± 0.45 µmol/L. VEGFR‐1 overexpressing cells also showed a significant increase of the basal Erk phosphorylation (Figure 6B). VEGFR‐1 overexpressing cells were more invasive compared with VEGFR‐1 negative control cells (Figure 6C,D). In addition, PlGF further stimulated ECM invasion by melanoma cells and the anti‐VEGFR‐1 mAb prevented PlGF effect (Figure 6C,D).

**Figure 7:**
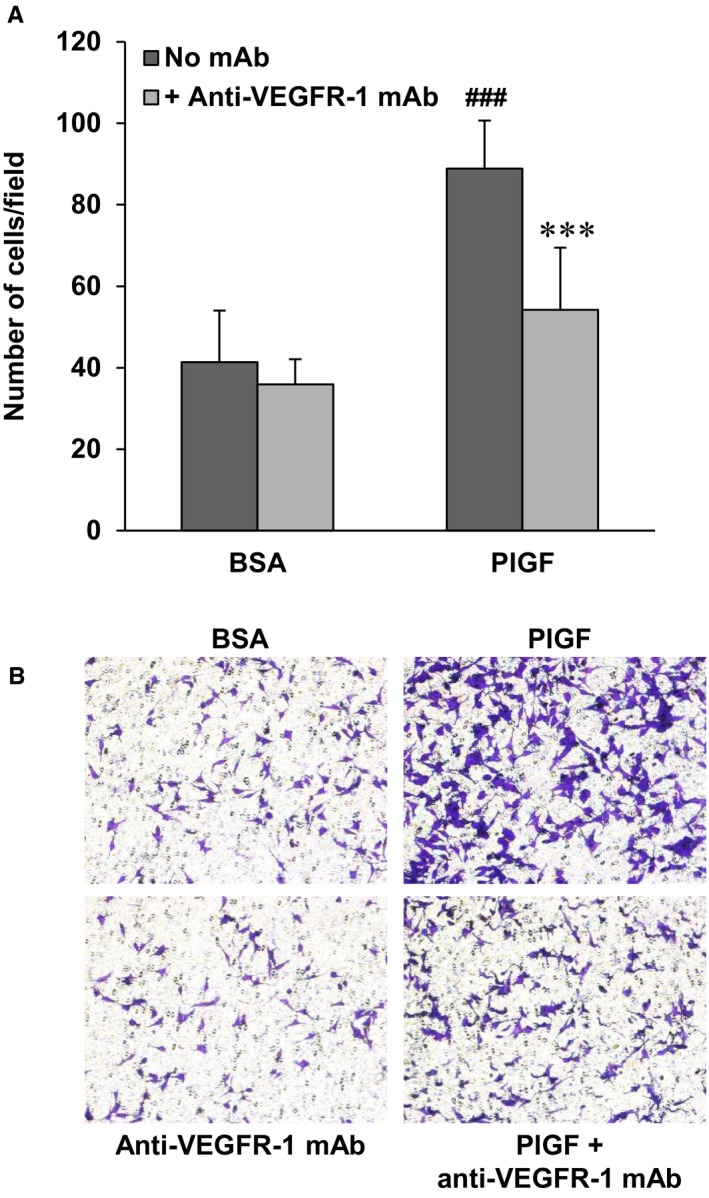
Inhibitory effect of the anti‐VEGFR‐1 mAb D16F7 on ECM invasion by A375‐VR melanoma cells in response to PlGF. A, ECM invasion of A375‐VR cells (2x10^5^ cells/chamber, 4 h incubation) induced by PlGF (50 ng/mL) in the absence or presence of 5 µg/mL D16F7 mAb. BSA, non‐stimulated cells. Histograms represent the arithmetic mean ± SD of invading cells/microscopic field from three independent experiments. Statistical analysis was performed by Kruskal‐Wallis followed by Dunn's test for multiple comparison: *P* < .001, *** A375 PlGF *vs* A375 PlGF + anti‐VEGFR‐1 mAb; ###, A375 PlGF *vs* A375 BSA. B, Photographs from a representative experiment out of three are shown (×100 magnification)

Finally, also in the case of the VEGFR‐1 proficient A375‐VR cells, D16F7 inhibited the stimulatory effects on ECM invasion of the VEGFR‐1 specific ligand PlGF (Figure 7).

## 4 DISCUSSION

The antitumour effects of vemurafenib are short‐lived and the majority of patients undergoing therapy present tumour relapse within few months after the beginning of treatment. Therefore, the characterization of the mechanisms contributing to vemurafenib resistance is essential in order to improve its long‐term efficacy and to identify next generation therapeutic strategies.

Adaptive tumour responses to BRAF‐targeted drugs are favoured by melanoma heterogeneity and lead to treatment failure. Acquired resistance mechanisms include the increased expression of several receptor tyrosine kinases, such as platelet‐derived growth factor receptor beta (PDGFRβ), insulin‐like growth factor‐1 receptor (IGF1R) and epidermal growth factor receptor (EGFR),^34^, ^35^, ^36^, ^37^ which activate signal transduction pathways alternative to BRAF. In the present study, we demonstrate for the first time that the up‐regulation of another receptor tyrosine kinase, VEGFR‐1, participates to the development of a resistant phenotype in melanoma. In fact, human melanoma cells rendered resistant to the BRAFi express higher VEGFR‐1 levels compared with their BRAFi‐sensitive counterparts. Moreover, transient silencing of the receptor in susceptible cells delays resistance occurrence, whereas in resistant cells down‐regulation of VEGFR‐1 increases sensitivity to the BRAFi. Consistently, enforced expression of VEGFR‐1, by stable gene transfection in receptor‐negative melanoma cells, markedly reduces sensitivity to vemurafenib. This finding is particularly relevant since we observed an increase in VEGF‐A secretion by resistant cells, as also reported for melanoma resistant to the BRAFi dabrafenib.^21^, ^22^, ^23^ Moreover, VEGFR‐1 is efficiently stimulated by its exclusive ligand PlGF that can be released by the tumour itself or by cells of the tumour microenvironment.

BRAF mutations can control processes such as invasion and metastasis, since BRAF down‐modulation reduces MAPK and MMP‐2 activities, and the invasive ability in a melanoma model.^38^ In particular, after the acquisition of vemurafenib resistance, melanoma cells showed reactivation of the MAPK pathway, including MEK and ERK proteins, and a pronounced CRAF phosphorylation.^32^, ^38^, ^39^, ^40^, ^41^ Our data also indicate that melanoma cells expressing VEGFR‐1 are more invasive than VEGFR‐1 deficient cells. Transition from a proliferative to an invasive phenotype has been implicated in the development of metastases.^32^ Actually, while M14 cells show proliferative properties and are not invasive, M14‐VR cells show a highly invasive behaviour and a significantly higher doubling time compared with M14 cells. Conversely, VEGFR‐1 positive A375‐VR and A375 cells are both characterized by an invasive phenotype and this might explain the lack of differences in their doubling times. In this context, and in agreement with previous studies, we recently reported that signalling induced by VEGFR‐1 activation further stimulates tumour invasiveness and results in ERK phosphorylation.^8^, ^30^, ^42^, ^43^ This receptor has been also correlated to cell survival and chemoresistance.^3^, ^10^, ^11^, ^44^ Moreover, increased VEGFR‐1 expression and/or up‐regulation of its specific ligand PlGF are considered mechanisms of tumour resistance to VEGF‐A targeting anti‐angiogenic therapies.^45^, ^46^, ^47^, ^48^

Overall, our results strongly support the hypothesis that VEGFR‐1 expression might contribute to the aggressive phenotype of melanoma cells resistant to vemurafenib. Actually, the recently described mAb D16F7, produced in our laboratories and that specifically inhibits VEGFR‐1, drastically reduces invasiveness of resistant cells. Interestingly, VEGFR‐1 blockade by D16F7 mAb reduces ECM invasion triggered by VEGF‐A and PlGF, supporting the hypothesis that up‐regulation of VEGFR‐1 might contribute to tumour progression and spreading of melanoma after acquisition of a drug‐resistant phenotype. Besides this activity, D16F7 mAb has also the ability to modify the tumour microenvironment at least at two different levels: hampering tumour‐associated angiogenesis and reducing melanoma infiltration by pro‐tumour macrophages. These D16F7 properties support its use in combination with BRAFi. It should also be noted that, since VEGFR‐1 does not play a relevant role in physiological angiogenesis in the adult, this combination is likely to result in increased therapeutic efficacy without causing additive toxicity.

In conclusion, our results strongly suggest that the selective VEGFR‐1 inhibition by D16F7 mAb might potentiate the effects of vemurafenib‐based therapies for melanoma treatment and counteract resistance development to this BRAFi.

## CONFLICT OF INTEREST

The authors confirm that there are no conflicts of interest.

## AUTHOR'S CONTRIBUTION

PML and GG designed the experiments and wrote the first draft of the manuscript. GG, PML, L.T, MLB and SDA critically reviewed and revised the article. MGA, CC, FR and MT performed the experiments. The final version of the paper was read and approved by all authors.

## Data Availability

The data that support the findings of this study are available from the corresponding author upon reasonable request.
